# Detection of SARS-CoV-2 IgA and IgG in human milk and breastfeeding infant stool 6 months after maternal COVID-19 vaccination

**DOI:** 10.21203/rs.3.rs-1950944/v1

**Published:** 2022-08-19

**Authors:** Lauren Stafford, Vivian Valcarce, Matthew Henry, Josef Neu, Leslie Parker, Mueller Martina, Valeria Vicuna, Taylor Gowen, Emilee Cato, Ivan Kosik, Jonathan Yewdell, Mark Atkinson, Nicole Cacho, Nan Li, Joseph Larkin

**Affiliations:** University of Florida; University of Florida; University of Florida; University of Florida; University of Florida; University of Florida; University of Florida; University of Florida; National Institutes of Health/National Institute of Allergy and Infectious Diseases; National Institutes of Health/National Institute of Allergy and Infectious Diseases; University of Florida; University of Florida; University of Florida; University of Florida

## Abstract

**Objective:**

Assess the presence, durability, and neutralization capacity of SARS-CoV-2-specific antibodies in breastfeeding infants’ stools, mother’s plasma, and human milk following maternal vaccination.

**Design:**

Thirty-seven mothers and 25 infants were enrolled between December 2020 and November 2021 for this prospective observational study. Human milk, maternal plasma, and infants’ stools were collected pre-vaccination and at periods up to 6 months following COVID-19 vaccine series initiation/completion. SARS-CoV-2 antibody levels and their neutralization capacities were assessed in collected samples.

**Results:**

SARS-CoV-2-specific IgA and IgG levels were higher in infant stool post-maternal vaccination amongst milk-fed compared to pre-COVID controls. Human milk and plasma SARS-CoV-2-specific IgA and IgG concentrations decreased over 6 months post-vaccination but remained higher than pre-vaccination levels. We observed improved neutralization capacity in milk antibodies over time.

**Conclusions:**

The presence of neutralizing SARS-CoV-2-specific antibodies in infant stool following maternal vaccination offers further evidence of the lasting transfer of these antibodies through breastfeeding and their protective effect.

## Introduction

The grievous consequences of COVID-19 infection are widely known. With the upsurge of new variants(1), we have witnessed how COVID-19 has affected the US (United States) population, including pregnant women(2) and children.(3)

Vaccination during pregnancy is known to generate functional anti-spike IgG antibodies in maternal circulation that are detectable in umbilical cord blood at birth and can protect the infant from COVID-19.(4–7) Further, recent studies have demonstrated that COVID-19 infection, as well as vaccination against COVID-19, induces secretion of neutralizing SARS-CoV-2 IgA and IgG in human milk (HM).(8–16) However, little is known about the transfer of neutralizing HM antibodies to breastfeeding infants.

HM has antimicrobial properties, evidenced by the decreased rate of infections and hospitalization among breastfeeding infants.(17) This can be attributed to molecules with immuno-protective functions within HM: immunoglobulins, lactoferrin, oligosaccharides, and cytokines, to name a few.(18) HM antibodies, in particular, sIgA, prevent the entry of microorganisms into the tissue by decreasing surface colonization and dampening penetration of potentially harmful organisms.(19)

SARS-CoV-2 relies on its main receptor, angiotensin-converting enzyme 2 (ACE2), to enter cells, which is abundantly present in the human epithelia of the lung, small intestine, and colon.(20, 21) Reports have shown that a notable proportion of patients with COVID-19 develop gastrointestinal symptoms and nearly half of patients have detectable SARS-CoV-2 RNA in their fecal samples.(22); further, airborne transport of infectious COVID-19 aerosols from wastewater have been linked to viral transmission.(23)

To protect against SARS-CoV-2 infection, the immune system must mount a robust and specific response. In addition to SARS-CoV-2-specific antibody concentration, antibody specificity, affinity, and neutralizing capacity are also necessary to elicit a proper immune response. As such, we hypothesize that there is a transfer of neutralizing HM SARS-CoV-2 specific IgA and IgG to the intestinal tract of breast-fed infants that can protect them from COVID-19 infection. Together our results provide interesting insight into the relationship between the durability of maternal antibodies subsequent to vaccination, and the potential protection of breastfed infants.

## Methods

### Study design

This prospective observational study was conducted at the University of Florida under institutional review board approval (#IRB202003255). Thirty-seven breastfeeding mothers were recruited before or after COVID-19 vaccination with Pfizer/BioNTech, Moderna, or Johnson & Johnson from December 2020 to November 2021. In June 2021, we added breastfeeding infant enrollment for stool collection. A total of 25 infants were enrolled from June to November 2021. Mothers contributed data at various time points up to 6 months after completing the series (8 mothers provided longitudinal HM and 7 provided plasma samples at pre-vaccination, post-1^st^ dose, 7–30 days, and 6 months post-vaccination). Inclusion criteria included breastfeeding women 18 years and older, pre-or-post-COVID-19 vaccination, and providing informed consent.

### Participants and procedures

Recruitment methods included the University of Florida’s institutional e-mail listserv and advertising flyers posted in University of Florida facilities and the surrounding community. Up to 37 mothers and 25 infants were recruited at different time points; 97 maternal serum, 102 maternal milk, and 32 infant stool samples were collected up to 6-time points relative to COVID-19 vaccination completion: pre-vaccination, 15–30 days after the first vaccine dose (for participants receiving two-dose mRNA vaccines), and then at 7–30 days, 60–75 days, 90–105 days, and 6 months following 2-dose vaccination series completion. Not all participants contributed samples at every listed collection time point (Supplemental Table 1). One mother withdrew from the study after the first sample collection; hence her sample was not analyzed.

Participants completed a questionnaire collecting maternal/infant demographics, medical and family history, and vaccination side effects upon agreeing to participate.

### Sample collection and processing

Maternal blood samples were collected via venipuncture or finger prick in ethylenediaminetetraacetic acid-coated (EDTA) tubes at the designated time points. Plasma was separated from cellular matter by centrifugation at 2000 g for 10 minutes at 4°C and then stored undiluted at −20°C.

For milk, mothers were instructed to express samples of 10–30 ml. Samples were stored at −20°C within 4 hours after collection. The samples were aliquoted into 2 mL tubes, then centrifuged at 500 g for 15 minutes at 4°C. Using a 21G needle, the aqueous layer was separated from the fat layer and placed in a clean tube. This aqueous layer was then centrifuged at 3000 g for 15 minutes at 4°C. The final aqueous layer was removed and stored undiluted at −20°C.

Stool samples were collected in diapers and dropped off either the same day or kept refrigerated overnight by participants before their collection appointments, then stored at −80°C until further processing. Stool samples were then diluted with 2 mL DPBS, vortexed vigorously to achieve a homogenized sample, and centrifuged at 1500 g for 20 minutes. The supernatant was then placed in a clean tube, vortexed, and centrifuged at 10 000 g for 10 minutes. Supernatant was removed and stored undiluted at −20°C.

SARS-CoV-2-specific IgA and IgG concentrations were measured in HM, plasma, and infant stool using previously-validated COVID-19 Human IgA and IgG ELISA kits (RayBiotech Life, Peachtree Corners, Georgia, USA).(10) SARS-CoV-2-specific IgA samples were run at 1:500 dilution in plasma. SARS-CoV-2-specific IgG samples were run at 1:1000 dilution in plasma. SARS-CoV-2-specific IgA samples were run at 1:3 dilution in HM. SARS-CoV-2-specific IgG samples were run undiluted (1:1) in HM. SARS-CoV-2-specific IgA and IgG samples were run undiluted (1:1) in infant stool. We used 10 pre-COVID-era and 1 maternal pre-vaccination infants stool samples as negative controls.

To determine the neutralization capability of SARS-CoV-2 antibodies, we used an assay containing vesicular stomatitis virus (VSV) expressing SARS-CoV-2 spike protein and green fluorescent protein (GFP) (VSV-gfp-SARS-CoV-2-S-gp) described in a previously published study.(24) Milk, plasma, or stool samples were mixed with VSV and later incubated with baby hamster kidney (BHK) cells expressing human ACE2 receptors. GFP frequency was measured by flow cytometry to quantify BHK-ACE2 cell infectivity. The raw GFP+ values of each sample were normalized to a scale of 0–100% infectivity using negative and positive controls (Supplemental Figure 2). The normalized values were then used to make non-linear regression curves of infectivity, providing IC50 values for each sample.

### Statistical analysis

The demographics and clinical features of the study sample were characterized via descriptive statistics. Medians with 25^th^ and 75^th^ percentiles (IQR1 and IQR3) were provided for log(10)-transformed SARS-CoV-2 specific milk, plasma, and stool IgA and IgG levels at all time points (Supplemental Table 1).

Mann Whitney U-tests (unpaired, nonparametric) were used to compare antibody concentrations in infant stool after mother’s COVID-19 vaccination vs. pre-COVID era infant stool samples across available time points; and IC50 in milk and plasma pre-vaccination vs. 7–30 days post-2nd dose and six months post-vaccination series completion. Spearman correlation was carried out to explore the relationship between log(10)-transformed SARS-CoV-2 specific IgA and IgG in milk, plasma, and infant stool at 6 months; and IgA and IgG in milk, plasma with IC50 in milk and plasma at three-time points (pre-vaccination, 7–30 days post-2nd dose, and six months post-vaccination series completion). Statistical analyses were performed using SPSS statistics software and GraphPad Prism 9 (GraphPad Software, La Jolla, CA, USA). All figures were created using GraphPad Prism 9.

## Results

Thirty-seven lactating women and 25 infants were enrolled in the study. Of those, 36 women and all 25 infants were included in the statistical analysis; 8 women completed longitudinal samples for HM and 7 for plasma at pre-vaccination, post-1^st^ dose, 15–30 days post-second dose, and 6 months after 2-dose vaccination series completion (Supplemental Figure 1). Pre-vaccination samples consisted of 25 HM and 16 plasma samples; 6-month samples included 16 HM and 19 plasma samples. Thirty-two infant’s stools samples were collected up to 6 months post-vaccination. The study population consisted primarily of White non-Hispanic women in their mid-30s and their infants with a median infant age of 10 months at enrollment (Table 1).

### 1. SARS-CoV-2 IgA and IgG are present in the stool of breastfeeding infants after maternal COVID-19 vaccination and can neutralize the COVID-19 virus

**Figure 1:**
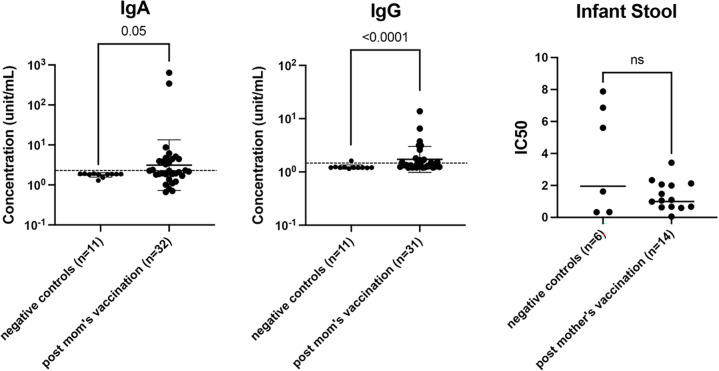
SARS-CoV-2 specific antibodies (IgA and IgG) and neutralization capacity (IC50) in infant stool up to 6 months after mother’s COVID-19 vaccination. IC50: the lower the IC50, the stronger the neutralization capability. Concentration (unit/mL) listed in log10 form. Both antibody concentration comparison and IC50 statistical calculations are measured using Mann Whitney unpaired, nonparametric test; the figure is shown as geometric mean and geometric SD.

We detected SARS-CoV-2 IgA and IgG in the stool of breastfeeding infants whose mothers received COVID-19 vaccination, with a statistically significant increase relative to negative controls (p = 0.05 and <0.0001 in IgA and IgG, respectively) (Figure 1).

We also observed an increase in stool neutralization capability after maternal COVID-19 vaccination. Neutralization capacity is measured by the half-maximal inhibitory concentration (IC50): the lower the IC50, the stronger the neutralization capability. The geometric mean IC50 for infant stool samples after mother’s vaccination was 0.99 compared to a geometric mean IC50 of 1.40 in negative control samples (Figure 1). Although not statistically significant (p = 0.4), neutralization capacity was consistently higher in mother-vaccinated infant stool samples. On average, there was a 30% increase in neutralization capacity in stool samples after mother’s vaccination compared to negative, pre-COVID controls.

### 2. SARS-CoV-2 IgA concentrations in human milk and plasma appear to decline over time but remain higher than pre-vaccination 6 months after.

**Figure 2:**
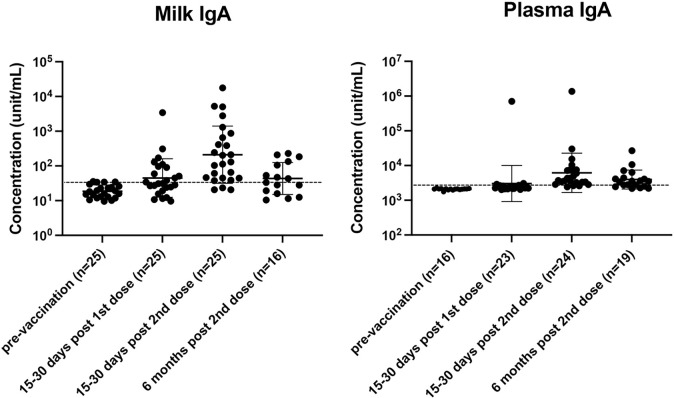
SARS-CoV-2 specific IgA in milk and plasma up to six months after COVID-19 vaccination. Concentration (unit/mL) is listed in log10 form. Figures are shown as geometric mean and geometric mean SD.

SARS-CoV-2-specific IgA concentrations in milk and plasma declined 6 months after vaccination, but levels remain higher than the pre-vaccination value (Figure 2). For milk, the median for log (10)-transformed SARS-CoV-2-IgA concentration was 1.3 unit/ml pre-vaccination versus 1.6 unit/ml six months post-vaccination. In plasma, the median for log (10)-transformed SARS-CoV-2-IgA concentrations was 3.3 unit/ml pre-vaccination, compared to 3.5 units/ml six months post-vaccination (Supplemental Table 1). Furthermore, the positivity rate decreased to 50% in milk and 74% in plasma at 6 months (Supplemental Table 1), from 85% at 7–30 days post-vaccination.(10)

Plasma IgA concentrations at six months post vaccination were positively correlated to plasma IgG concentrations (p-value=0.02, R=0.55) (Supplemental Table 2).

### 3. SARS-CoV-2 IgG concentration appears to decline in plasma and to a lesser degree in human milk 6 months after COVID-19 vaccination

**Figure 3:**
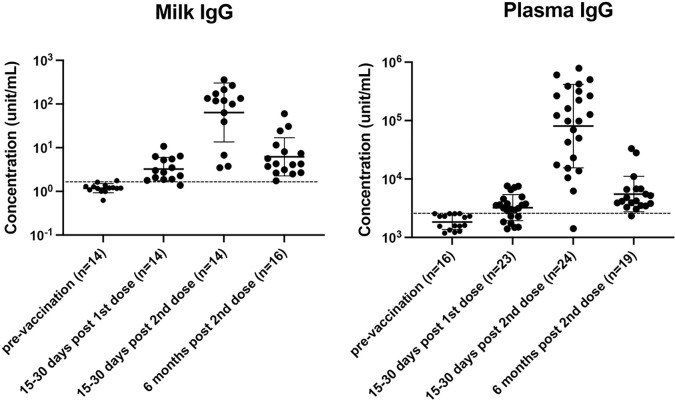
SARS-CoV-2 specific IgG in milk and plasma up to six months after COVID-19 vaccination. Concentration (unit/mL) is listed in log10 form. Figures are shown as geometric mean and geometric mean SD.

Although SARS-CoV-2 IgG decreased in HM 6 months after vaccination, values remained higher than pre-vaccination levels (Figure 3). Median log (10)-transformed SARS-CoV-2-IgG concentrations in HM pre-vaccination were 0.08 units/ml, versus 0.6 units/ml six months post-vaccination (Supplemental Table 1). In plasma, we observed a steep decline in SARS-CoV-2 IgG six months after vaccination compared to immediately post second dose; however, the positivity rate remained above 90% after 6 months, with 100% above the positive cutoff in milk and 95% above the positive cutoff in plasma. (Supplemental Table 1).

At six months post-vaccination, plasma IgG and milk IgG concentrations were positively correlated (p=0.074, R=0.46) (Supplemental Table 2). For both milk and plasma, we noticed an overall decline in SARS-CoV-2 antibody levels at six months compared to 7–30 days post-vaccination, with a more pronounced decrease in IgG vs. IgA. However, it should be noted that the increase in IgG after vaccination was more pronounced than IgA.

### 4. Human milk neutralizing activity

**Figure 4:**
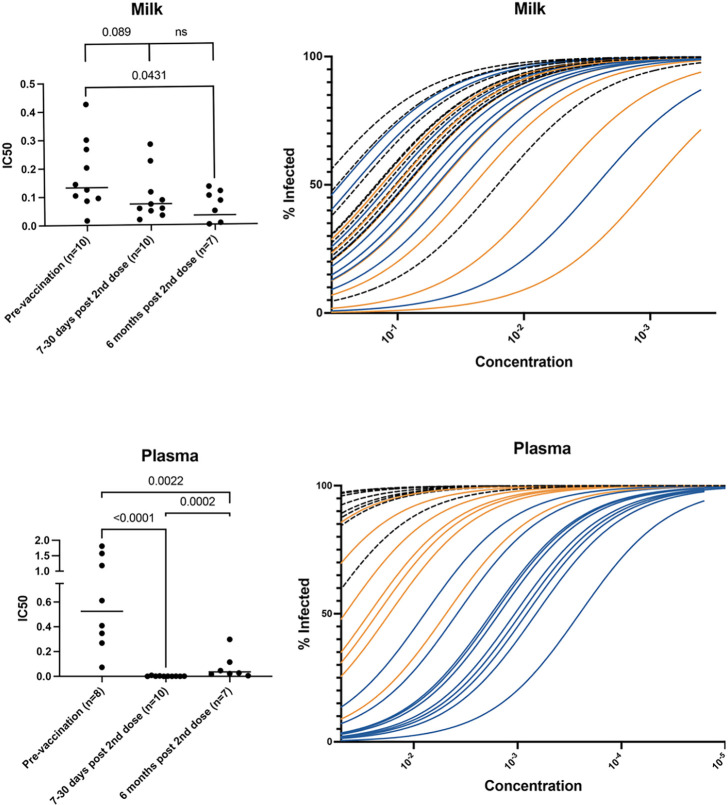
Neutralization of VSV-gfp-SARS-CoV-2-S in milk (top) and plasma (bottom). In milk and plasma, neutralization is compared pre-vaccination, 7–10 days post 2^nd^ dose, and six months post vaccination using the half-proximal inhibitory concentration (IC50). IC50 statistical calculations are measured using Mann Whitney unpaired, nonparametric tests. Figures are shown as geometric mean. Line graphs represent non-linear regression models of inhibition at pre-vaccination (dotted, black line), 7–10 days post 2^nd^ dose (solid, blue line), and six months post vaccination (solid, orange line).

Human milk neutralized viral binding of the pseudovirus VSV-gfp-SARS-CoV-2-S-gp to the BHK21-ACE2 cell line *in vitro* pre- and post-COVID-19 vaccination. However, the highest neutralization capability was observed 6 months after vaccination. The geometric mean IC50 (indicative of viral adherence to cells) for HM pre-vaccination was 0.13, compared to 0.03 six months post-vaccination (Figure 4). The increase in HM neutralization capability after vaccination was statistically significant with a p-value of 0.04. Neutralization capacity increased by 75% six months after COVID-19 vaccination completion compared to pre-vaccination milk. This work supports current, published data that shows milk can neutralize SARS-CoV-2 after COVID-19 mRNA vaccination.(8,25)

### 5. Plasma neutralizing activity

We detected a statistically significant increase in plasma neutralization capability after COVID-19 vaccination (p < 0.0001) (Figure 4). Before vaccination, the geometric mean IC50 in plasma was 0.5. The highest neutralization occurred 7–30 days after the second vaccine dose (IC50=0.001) and diminished six months after vaccination (IC50= 0.036) while remaining above pre-vaccination levels. Neutralization capacity increased by 99% in plasma immediately post COVID-19 vaccination completion and remained statistically, significantly higher than pre-vaccination levels six months after vaccination (p=0.002). The decline in neutralization is also observed in other studies that show neutralization in blood peaks roughly two weeks following the second dose but falls in vaccinated participants over time.(26,27)

Plasma neutralization (IC50) was statistically significantly correlated to plasma IgA concentrations 7–30 days post 2^nd^ dose (R=−0.79, p=0.009) (Supplemental Table 3). This means the higher the plasma SARS-CoV-2 IgA concentration, the higher the plasma neutralization.

## Discussion

Infants are born with an immature immune system, and it has been well-established that breastfeeding protects them against respiratory infections.(17–19) Prior studies have shown a significant increase in neutralizing SARS-CoV-2 specific antibodies in HM after COVID-19 vaccination or infection, suggesting the transfer of these antibodies to breastfeeding infants.(10,11,14–16,28) Our study is among the first to demonstrate the presence of neutralizing SARS-CoV-2 antibodies in breastfeeding infants’ stools with an average 30% increase in infant stool neutralization capabilities following maternal SARS-CoV-2 vaccination. Despite not being statistically significant, likely secondary to a small sample size, our results further support the maternal/infant passive transfer of SARS-CoV-2 antibodies through HM and their protective effect on breastfeeding infants.

The human small bowel and colon are rich in ACE-2 receptors, and studies have shown that SARS-CoV-2 preferentially affects cells with higher expression of ACE-2.(21) Furthermore, the pediatric population can shed SARS-CoV-2 RNA for up to several weeks in the stool after confirmed respiratory clearance.(29) These observations have led to the standing hypotheses that injury to intestinal cells contributes to the gastrointestinal symptoms of COVID-19 infection and that gastrointestinal cells may be an entry point for the virus.(22) We hypothesize that stool SARS-CoV-2 antibodies bind to ACE-2 receptors and block viral entry through the intestinal tract.

It should be noted that HM was able to neutralize the pseudovirus *in vitro* even before vaccination. This can likely be attributed to the extensive array of immunoprotective molecules within HM (immunoglobulins, IgA secretory component, lactoferrin, oligosaccharides, blood-derived leukocytes) and is in line with the known protection that breastfeeding offers to infants against numerous other respiratory viruses.(17)(30) Furthermore, once vaccination is administered to breastfeeding mothers, we observe a progressive increase in HM neutralization capability over time despite a decrease in antibody levels; this could result from the specialization and affinity maturation of secreted antibodies in HM.

These results lead us to hypothesize that secreted HM SARS-CoV-2 IgA and IgG may serve to neutralize the virus and offer immunologic protection to breastfeeding infants, potentially helping prevent SARS-CoV-2 infection and reducing the severity of associated symptoms. Further studies with larger populations are needed to assess the clinical significance of these findings.

### Limitations:

This study is among the first to suggest an effective transfer of HM SARS-CoV-2 IgA and IgG to breastfeeding infants’ gastrointestinal tract. There are limitations to some of the results. First, there was a small sample size and limited diversity in this cohort of mothers. Although participants contributed data at various time points, no subjects contributed on all occasions. Furthermore, whether infants or mothers might have had a previously undiagnosed COVID infection, and how this may have interfered with results, cannot be known. The mean age of our infant subjects was 10 months, with many of them having solid food their main source of nutrition and breastfeeding as supplementation; this in combination with small sample size might have interfered with the stool antibodies concentration and neutralization capacity results, since exclusively breastfeeding infants may have higher antibodies levels.

## Supplementary Material

Supplement 1

Supplement 2

Supplement 3

Supplement 4

## Figures and Tables

**Table 1.** Study Participants Characteristics^a^

**Maternal characteristics (*n* = 36)**	***N*(%) or mean ± standard deviation** ^a^
Age (years)	33.5 ± 3.9
Race	
White	35 (97)
Asian	1 (3)
Ethnicity	
Non-Hispanic	25 (69)
Hispanic	7 (19)
Not disclosed	4 (11)
Body mass index (kg/m^2^)^b,c^	24.3 ± 3.9
Normal/healthy weight	22 (61)
Overweight	9 (25)
Obese	4 (11)
History of allergies^c^	9 (25)
History of asthma^c^	4 (11)
History of inadequate immune response to vaccine^c^	3 (8)
History of known COVID-19 infection^d^	1 (3)
Family history of cancer^c^	19 (53)
Family history of autoimmune disorder^c^	3 (8)
Antibiotic use in 6 months before enrollment^c^	11 (31)
Time postpartum (months)	5.2 ± 6.2
Decreased human milk supply after COVID vaccine^c^	
No	27 (84)
Yes	2 (6)
Transient	3 (9)
Vaccine brand^c^	
Moderna	13 (36)
Pfizer	20 (56)
Johnson and Johnson	2 (6)
**Infant characteristics (*n* = 25)**	***N*(%) or mean ± standard deviation** ^a^
Infant gender	
Female	11 (44)
Male	14 (56)
Infant age at the time of stool collection (months)	10 ± 6
Number of infants in each age group ^e^	
<6 months	6 (23)
6–12 months	12 (46)
13–24 months	6 (23)
>24 months	2 (8)

a Categorical data are given as the number of participants and, in parentheses, the percentage of the total. Continuous data are provided as means ± standard deviations.

b Definitions put forth by the U.S. Centers for Disease Control and Prevention were used for body mass index categories.^16^

c Missing data from 1 subject.

d Pre-vaccination sample data unavailable from a subject with known prior COVID-19 infection.

e Age at time of stool sample collection; N = 26 due to one infant being in different age groups on dates of different sample collections.
