# Supplementary material for: Detection of SARS-CoV-2 IgA and IgG in human milk and breastfeeding infant stool 6 months after maternal COVID-19 vaccination
Source: Res Sq. 2022 Aug 19:rs.3.rs-1950944. Preprint. [Version 1] doi: 10.21203/rs.3.rs-1950944/v1 (PMC9413712; doi:10.21203/rs.3.rs-1950944/v1)
Supplement: Supplement 1 [file SupplementalMaterialtablesJoP.docx]

**Supplemental Material:**

**Supplemental Table 1. Log(10)-transformed SARS-CoV-2 specific IgA and IgG in maternal milk, plasma and infant stool (infant)**

| **Log(10)-transformed SARS-CoV-2 specific IgA and IgG** | **N** | **Median [IQR1; IQR3]** | **Percent Positive** |
| --- | --- | --- | --- |
| **Milk IgA** |  |  |  |
| Pre-vaccination | 25 | 1.3 [1.1,1.5] | 2/25 (8%) |
| 15-30 days post first dose | 25 | 1.5 [1.4,2.0] | 11/25 (44%) |
| 7-30 days post second dose | 25 | 2.1 [1.7,2.8] | 22/25 (88%) |
| 60-75 days post second dose | 5 | 1.9 [1.8,2.5] | 4/5 (80%) |
| 90-105 days post second dose | 9 | 1.8 [1.6,2.0] | 6/9 (67%) |
| 6 months post second dose | 16 | 1.6 [1.3,2.0] | 8/16 (50%) |
| **Milk IgG** |  |  |  |
| Pre-vaccination | 14 | 0.08 [0.04,0.11] | 1/14 (7%) |
| 15-30 days post first dose | 14 | 0.5 [0.3,0.7] | 13/14 (93%) |
| 7-30 days post second dose | 14 | 2.1 [1.6,2.2] | 14/14 (100%) |
| 60-75 days post second dose | 5 | 1.8 [1.5,2.0] | 5/5 (100%) |
| 90-105 days post second dose | 9 | 1.1 [0.8,1.4] | 9/9 (100%) |
| 6 months post second dose | 16 | 0.6 [0.5,0.9] | 16/16 (100%) |
| **Plasma IgA** |  |  |  |
| Pre-vaccination | 16 | 3.3 [3.32,3.33] | 0/16 (0%) |
| 15-30 days post first dose | 23 | 3.4 [3.3,3.4] | 4/23 (17%) |
| 7-30 days post second dose | 24 | 3.6 [3.5,3.9] | 21/24 (88%) |
| 60-75 days post second dose | 4 | 4.5 [4.1,4.9] | 4/4 (100%) |
| 90-105 days post second dose | 9 | 3.8 [3.5,4.1] | 7/9 (78%) |
| 6 months post second dose | 19 | 3.5 [3.4,3.6] | 14/19 (74%) |
| **Plasma IgG** |  |  |  |
| Pre-vaccination | 16 | 3.27 [3.2,3.4] | 0/16 (0%) |
| 15-30 days post first dose | 23 | 3.5 [3.4,3.6] | 16/23 (70%) |
| 7-30 days post second dose | 24 | 5.0 [4.3,5.4] | 23/24 (96%) |
| 60-75 days post second dose | 4 | 5.5 [5.4,5.6] | 4/4 (100%) |
| 90-105 days post second dose | 9 | 4.1 [4.0,5.0] | 9/9 (100%) |
| 6 months post second dose | 19 | 3.6 [3.6,3.8] | 18/19 (95%) |
| **Infant stool IgA** |  |  |  |
| Pre-vaccination | 11 | 0.27 [0.26,0.27] | 0/11 (0%) |
| 15-30 days post first dose | 1 | 0.4 [-] | 0/1 (0%) |
| 7-30 days post second dose | 3 | 0.6 [0.46,0.65] | 2/3 (67%) |
| 60-75 days post second dose | 3 | 0.26 [0.2,0.4] | 1/3 (33%) |
| 90-105 days post second dose | 11 | 0.3 [0.28,0.36] | 2/11 (18%) |
| 6 months post second dose | 13 | 0.5 [0.1,0.7] | 7/13 (54%) |
| **Infant stool IgG** |  |  |  |
| Pre-vaccination | 11 | 0.08 [0.08; 0.09] | 1/11 (9%) |
| 15-30 days post first dose | 1 | 0.08 [-] | 0/1 (0%) |
| 7-30 days post second dose | 2 | 0.45 [0.27,0.63] | 1/2 (50%) |
| 60-75 days post second dose | 3 | 0.22 [0.21,0.23] | 3/3 (100%) |
| 90-105 days post second dose | 11 | 0.12 [0.1,0.18] | 3/11 (27%) |
| 6 months post second dose | 13 | 0.12 [0.1,0.26] | 5/13 (38%) |

**Supplemental Table 2. Correlations between log(10)-transformed SARS-CoV-2 specific IgA and IgG in milk, plasma, and infant stool at 6 months post vaccination**

| **Log(10)-transformed SARS-CoV-2 specific IgA and IgG)** | **Maternal Plasma IgG** | **Maternal Milk IgG** | **Infant Stool IgG** | **Maternal Milk IgA** | **Maternal Plasma IgA** | **Infant Stool IgA** |
| --- | --- | --- | --- | --- | --- | --- |
| **Maternal Milk IgA** | 0.09  P=0.74 | 0.03  P=0.90 | 0.45  P=0.14 | - | 0.14  P=0.60 | 0.09  P=0.76 |
| **Maternal Plasma IgA** | 0.55  P=0.017 | 0.44  P=0.09 | 0.28  P=0.37 | 0.14  P=0.60 | - | -0.05  P=0.88 |
| **Infant Stool IgA** | -0.21  P=0.50 | 0.06  P=0.85 | 0.11  P=0.74 | 0.09  P=0.76 | -0.05  P=0.88 | - |
| **Maternal Milk IgG** | 0.46  P=0.074 | - | - | - | - | - |
| **Maternal Plasma IgG** | - | 0.46  P=0.074 | - | - | - | - |
| **Infant Stool IgG** | 0.33  P=0.29 | 0.36  P=0.25 | - | - | - | - |

**Supplemental Table 3. Spearman correlation between log(10)-transformed SARS-CoV-2 specific IgA and IgG in milk, plasma and IC50 in milk and plasma at three time points (pre-vaccination, 7-10 days post 2^nd^ dose, and six months post vaccine completion)**

|  | **Pre-vaccination** | | **Post 2^nd^ dose IC50** | | **Six months post IC50** | |
| --- | --- | --- | --- | --- | --- | --- |
| Log(10)-transformed SARS-CoV-2 specific IgA and IgG) | **Milk** | **Plasma** | **Milk** | **Plasma*** | **Milk** | **Plasma** |
| **Milk IgA** | 0.2  P=0.58 | - | -0.54  P=0.11 | **-** | -0.25  P=0.59 | - |
| **Plasma IgA** | - | 0.52  P=0.20 | - | -0.79  P=0.0088 | - | 0.21  P=0.66 |
| **Milk IgG** | 0.4  P=0.75 | - | -0.2  P=0.92 | - | 0  P>0.99 | - |
| **Plasma IgG** | - | -0.09  P=0.85 | - | -0.41  P=0.25 | - | -0.18  P=0.71 |

*Negative correlation between antibody concentrations and IC50 7-10 days post 2^nd^ dose = Higher antibody concentration, higher neutralization
