# Supplementary material for: Detection of SARS-CoV-2 IgA and IgG in human milk and breastfeeding infant stool 6 months after maternal COVID-19 vaccination
Source: Res Sq. 2022 Aug 19:rs.3.rs-1950944. Preprint. [Version 1] doi: 10.21203/rs.3.rs-1950944/v1 (PMC9413712; doi:10.21203/rs.3.rs-1950944/v1)
Supplement: Supplement 2 [file SupplementalMaterialsMethodsJoP.docx]

**Supplemental Materials**

**Assay protocol**

ELISA was performed for SARS-CoV-2-specific IgA and IgG in human milk, plasma, and infant stool using COVID-19 Human IgA and IgG ELISA Kit (RayBiotech Life, Peachtree Corners, Georgia, USA) . We validated the RayBiotech COVID-19 Human IgA ELISA Kit for human milk using a 1:3 dilution series (1:3, 1:9, 1:27, 1:81, and 1:243) and used this dilution series to establish an antibody titer in a previously published study.(10) All ELISA samples were ran in duplicate.

SARS-CoV-2-specific IgA samples were diluted 1:3 in human milk. SARS-CoV-2 specific IgA samples were diluted 1:500 in plasma. SARS-CoV-2-specific IgG samples were run undiluted (1:1) in human milk. SARS-CoV-2 specific IgG samples were diluted 1:1000 in plasma. SARS-CoV-2 specific IgA and IgG samples were run undiluted (1:1) in infant stool.

**Neutralization assay protocol**

Neutralization was adapted from a previously published protocol.(24) Pseudotyped VSV-gfp-SARS-CoV-2-sgp was used to test the neutralization capacity of SARS-CoV-2 specific antibodies in human milk, plasma, and infant stool before and after COVID-19 vaccination. Human milk (3-fold dilution series), plasma (5-fold dilution series), and infant stool samples (2-fold dilution series) were serially diluted and were incubated at 37°C with the pseudovirus for 1 hour. The virus/antibody mixture was subsequently added to infection-competent BHK-21 cells expressing the ACE-2 receptor to simulate natural infection (plated at 40 000 cells/100uL) and incubated overnight at 37°C. The next day, GFP fluorescence positivity was measured on the BD Accuri to quantify the neutralization capacity of the sample-derived antibodies. Sample values were normalized by the value of control samples (BHK-ACE2 cells with or without VSV-gfp-SARS-CoV-2-S-gp added). (Supplemental Figure 2)

**BHK21-ACE2 cell maintenance**

A replicative VSVdG-SARS2-SgpFL pseudovirus and a permissive BHK21 cell line expressing human ACE2 receptor were kindly provided by Dr. Yewdell and Dr. Kosik (NIH/NIAID) and used for the neutralization assay.(24)BHK-ACE2 cell line was maintained in DMEM supplemented with 8% FBS and incubated at 37°C, 5% CO_2_.

**Viral propagation**

Pseudovirus was propagated as previously shown with modifications. (24)BHK-ACE2 were seeded and allowed to grow to 95% confluency. Once 95% confluency was reached, 50µL of VSVdG-SARS2-SgpFL pseudovirus viral stock in 3 mL DMEM containing 8% FBS was added to the flask. The flask was gently rocked for one hour for even inoculum distribution, followed by inoculum aspiration and the addition of media (10 ml). Once 95% of the monolayer was depleted (48-72 hours post-infection), the supernatant was collected and centrifuged (4500 RPM) for ten minutes. The supernatant was transferred to a new centrifuge tube and pellet resuspended in 3 mL DPBS. The pellet was subsequently disrupted for four consecutive freezes, thaws, and then sonication cycles. Following centrifugation at 4500 RPM for 10 minutes, the disrupted pellet contents were combined with previously saved supernatant, aliquoted into 0.5 mL volumes, and stored at -80°C for future use.
