# Supplementary figures and images for: Detection of SARS-CoV-2 IgA and IgG in human milk and breastfeeding infant stool 6 months after maternal COVID-19 vaccination
Source: Res Sq. 2022 Aug 19:rs.3.rs-1950944. Preprint. [Version 1] doi: 10.21203/rs.3.rs-1950944/v1 (PMC9413712; doi:10.21203/rs.3.rs-1950944/v1)

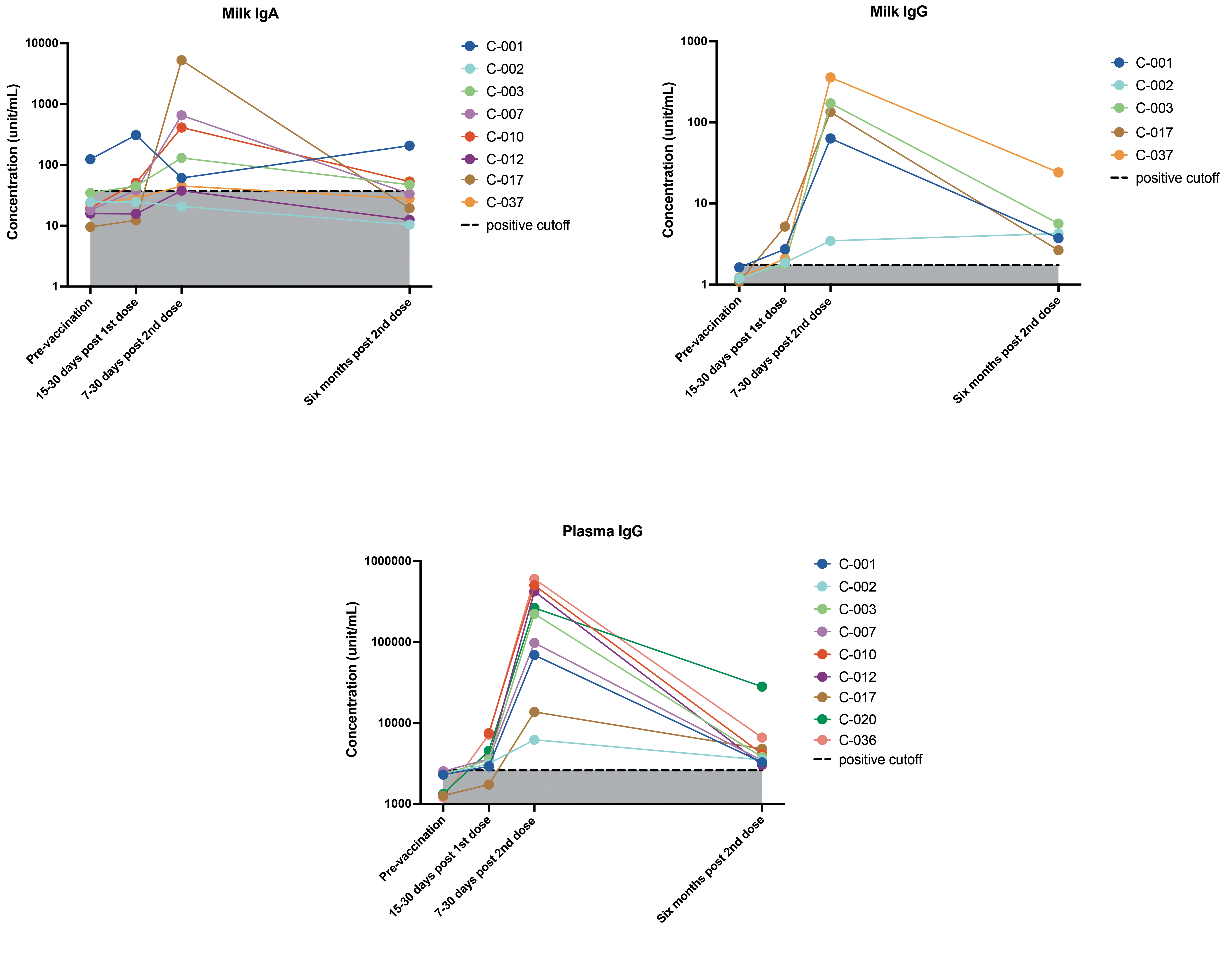

Supplement: Supplement 3 [file SuppFig1.png]

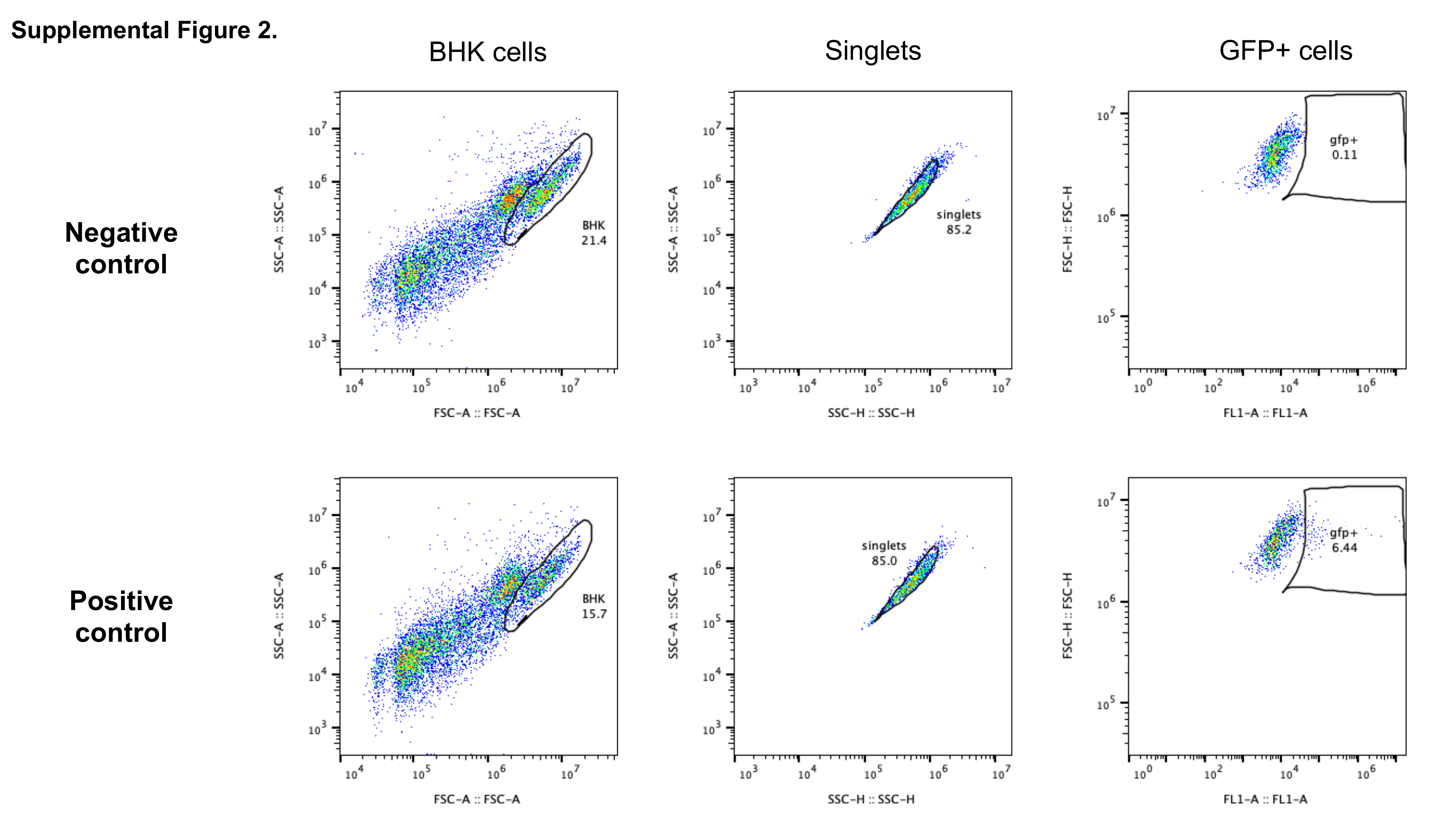

Supplement: Supplement 4 [file SupplFig2.png]
